# Assessment of NO_2_ observations during DISCOVER-AQ and KORUS-AQ field campaigns

**DOI:** 10.5194/amt-13-2523-2020

**Published:** 2020-05-19

**Authors:** Sungyeon Choi, Lok N. Lamsal, Melanie Follette-Cook, Joanna Joiner, Nickolay A. Krotkov, William H. Swartz, Kenneth E. Pickering, Christopher P. Loughner, Wyat Appel, Gabriele Pfister, Pablo E. Saide, Ronald C. Cohen, Andrew J. Weinheimer, Jay R. Herman

**Affiliations:** 1 NASA Goddard Space Flight Center, Greenbelt, MD 20771, USA; 2 Science Systems and Applications, Inc., Lanham, MD 20706, USA; 3 Universities Space Research Association, Columbia, MD 21046, USA; 4 Goddard Earth Sciences Technology and Research, Morgan State University, Baltimore, MD 20251, USA; 5 Johns Hopkins University, Applied Physics Laboratory, Laurel, MD 20723, USA; 6 Department of Atmospheric and Oceanic Science, University of Maryland, College Park, MD 20742, USA; 7 NOAA Air Resources Laboratory, College Park, MD 20740, USA; 8 Environmental Protection Agency, Research Triangle Park, NC 27709, USA; 9 National Center for Atmospheric Research, Boulder, CO 80301, USA; 10 Department of Atmospheric and Oceanic Sciences, and Institute of the Environment and Sustainability, University of California, Los Angeles, CA 90095, USA; 11 Department of Chemistry and Department of Earth and Planetary Science, University of California, Berkeley, CA 94720, USA; 12 Joint Center for Earth Systems Technology, University of Maryland Baltimore County, Baltimore, MD 21250, USA

## Abstract

NASA’s Deriving Information on Surface Conditions from Column and Vertically Resolved Observations Relevant to Air Quality (DISCOVER-AQ, conducted in 2011–2014) campaign in the United States and the joint NASA and National Institute of Environmental Research (NIER) Korea–United States Air Quality Study (KORUS-AQ, conducted in 2016) in South Korea were two field study programs that provided comprehensive, integrated datasets of airborne and surface observations of atmospheric constituents, including nitrogen dioxide (NO_2_), with the goal of improving the interpretation of spaceborne remote sensing data. Various types of NO_2_ measurements were made, including in situ concentrations and column amounts of NO_2_ using ground- and aircraft-based instruments, while NO_2_ column amounts were being derived from the Ozone Monitoring Instrument (OMI) on the Aura satellite. This study takes advantage of these unique datasets by first evaluating in situ data taken from two different instruments on the same aircraft platform, comparing coincidently sampled profile-integrated columns from aircraft spirals with remotely sensed column observations from ground-based Pandora spectrometers, intercomparing column observations from the ground (Pandora), aircraft (in situ vertical spirals), and space (OMI), and evaluating NO_2_ simulations from coarse Global Modeling Initiative (GMI) and high-resolution regional models. We then use these data to interpret observed discrepancies due to differences in sampling and deficiencies in the data reduction process. Finally, we assess satellite retrieval sensitivity to observed and modeled a priori NO_2_ profiles. Contemporaneous measurements from two aircraft instruments that likely sample similar air masses generally agree very well but are also found to differ in integrated columns by up to 31.9 %. These show even larger differences with Pandora, reaching up to 53.9 %, potentially due to a combination of strong gradients in NO_2_ fields that could be missed by aircraft spirals and errors in the Pandora retrievals. OMI NO_2_ values are about a factor of 2 lower in these highly polluted environments due in part to inaccurate retrieval assumptions (e.g., a priori profiles) but mostly to OMI’s large footprint (> 312 km^2^).

## 1 Introduction

Nitrogen dioxide (NO_2_) plays an important role in the troposphere by altering ozone production and OH radical concentration (Murray et al., 2012, 2014). It is one of the six United States Environmental Protection Agency (EPA) criteria pollutants because of its adverse health effects on humans (WHO, 2013). Major sources of nitrogen oxides (NO_*x*_=NO + NO_2_) in the troposphere include combustion, soil, and lightning. As a trace gas with a relatively short lifetime, NO_2_ is usually confined to a local scale with respect to its source and therefore exhibits strong spatial and temporal variations, leading to difficulties in comparing NO_2_ observations by methods with different atmospheric sampling.

Due to its distinct absorption features at ultraviolet–visible (UV–Vis) wavelengths, atmospheric NO_2_ is observable from ground- and space-based remote sensing instruments. In particular, space-based measurements of tropospheric column NO_2_ have been widely used to study spatial and temporal patterns (e.g., Beirle et al., 2003; Richter et al., 2005; Boersma et al., 2008; Lu and Streets, 2012; Wang et al., 2012; Hilboll et al., 2013; Russell et al., 2010, 2012; Duncan et al., 2013; Lin et al., 2015) as well as long-term trends (e.g., van der A et al., 2008; Lamsal et al., 2015; Krotkov et al., 2016), and to infer NO_*x*_ sources (e.g., Jaeglé et al., 2005; van der A et al., 2008; Bucsela et al., 2010; de Wildt et al., 2012; Lin, 2012; Ghude et al., 2010; Ghude et al., 2013a; Mebust and Cohen, 2013; Pickering et al., 2016) and top-down NO_*x*_ emissions (e.g., Martin et al., 2003; Konovalov et al., 2006; Zhao and Wang, 2009; Lin et al., 2010; Lamsal et al., 2011; Ghude et al., 2013b; Vinken et al., 2014; Schreier et al., 2015; Cooper et al., 2017; Miyazaki et al., 2017; Liu et al., 2018). These observations have also been often used to assess chemical mechanisms (e.g., Martin et al., 2002; van Noije et al., 2006; Lamsal et al., 2008; Kim et al., 2009; Herron-Thorpe et al., 2010; Huijnen et al., 2010) and to infer the lifetime of NO_*x*_ (e.g., Schaub et al., 2007; Lamsal et al., 2010; Beirle et al., 2011) in chemical transport models (CTMs). Surface NO_2_ concentrations (Lamsal et al., 2008, 2014; Novotny et al., 2011; Bechle et al., 2013) and NO_*x*_ deposition flux (Nowlan et al., 2014; Geddes and Martin, 2017) can also be estimated using satellite NO_2_ observations. As the accuracy of any application of satellite data largely depends on the data quality, validation of satellite NO_2_ observations is necessary.

A number of validation studies of space-based tropospheric NO_2_ columns have been conducted using independent NO_2_ observations from airborne in situ mixing ratio measurements (e.g., Boersma et al., 2008; Bucsela et al., 2008; Hains et al., 2010; Lamsal et al., 2014), ground-based total column (e.g., Pandora instrument; Herman et al., 2009) and tropospheric (MAX-DOAS instrument; e.g., Vlemmix et al., 2010; Irie et al., 2012) column measurements, and airborne high-resolution differential optical absorption spectroscopy (DOAS) measurements (Lamsal et al., 2017; Nowlan et al., 2018). Most validation studies utilizing in situ and ground-based observations have reported that satellite measurements tend to underestimate tropospheric NO_2_ columns, especially over highly polluted areas (e.g., Hains et al., 2010). Intrinsic limits of space-based measurements, however, pose a challenge in comparisons between satellite, in situ, and ground-based measurements due to differences in representativeness. As stated above, NO_2_ usually exhibits very sharp spatial gradients (tens of meters to kilometers). In contrast, the spatial resolution of satellite measurements is too coarse (tens of kilometers) to capture the fine spatial features of tropospheric NO_2_ abundance. Therefore, it is important to recognize and account for the spatial variability while comparing satellite data with ground-based and in situ observations.

While the intrinsic resolution of satellite observations cannot be altered, there are ways to improve the derived satellite data products. The fidelity of the retrieved NO_2_ product is dependent on the assumptions (e.g., NO_2_ vertical profile shape, surface reflectivity) made in the retrieval algorithm. Some of the input parameters are available at much coarser resolution than the spatial resolution of OMI, introducing spatially (e.g., rural-to-urban) varying retrieval biases. Several studies show that the use of high-resolution NO_2_ profiles results in significant improvements in retrievals (e.g., Russell et al., 2012; Lin et al., 2014; Lamsal et al., 2014; McLinden et al., 2014; Laughner et al., 2016, 2019; Goldberg et al., 2017). Deficiencies in model distributions of NO_2_ may be identified and improved through rigorous evaluation with independent data, such as the suite of data collected during the Deriving Information on Surface Conditions from Column and Vertically Resolved Observations Relevant to Air Quality (DISCOVER-AQ) campaign deployments.

In this paper, we use comprehensive, integrated datasets of NO_2_ gathered from surface, aircraft, and space instruments during NASA DISCOVER-AQ and the NASA and National Institute of Environmental Research (NIER) Korea–United States Air Quality Study (KORUS-AQ) together with NO_2_ model simulations to address questions regarding retrieval accuracy. We describe the datasets in Sect. 2.1 and the models in Sect. 2.2. As an example, we focus on the NASA Standard NO_2_ Product from OMI onboard the Aura satellite and conduct retrieval studies using the algorithm as discussed in Sect. 2.3, but the approaches discussed here could be applied to similar products as well. Results are presented in Sect. 3.

## 2 Observations and chemical transport models

### 2.1 NO_2_ observations during DISCOVER-AQ and KORUS-AQ field campaigns

DISCOVER-AQ (https://www-air.larc.nasa.gov/missions/discover-aq/, last access: 5 September 2019) and KORUS-AQ (https://www-air.larc.nasa.gov/missions/korus-aq/, last access: 5 September 2019) were field study programs that provided comprehensive, integrated datasets of airborne and surface observations relevant to the diagnosis of surface air quality conditions from space. DISCOVER-AQ was a part of the NASA Earth Venture program and conducted four field deployments in Maryland (MD), California (CA), Texas (TX), and Colorado (CO) that covered different seasons and pollution regimes. KORUS-AQ was an international cooperation field study program conducted in the Republic of Korea (South Korea), sponsored by NASA and the South Korean government through the NIER. Table 1 summarizes the campaign locations and periods for the two field campaigns.

The primary objectives of DISCOVER-AQ and KORUS-AQ included (1) exploring the relationship between air quality at the surface and the tropospheric columns that can be derived from satellite orbit, (2) examining the diurnal variation of these relationships, and (3) characterizing the scales of variability relevant to the model simulation and remote observation of air quality. To accomplish these objectives, an observing strategy was designed to carry out systematic and concurrent in situ and remote sensing observations from a network of ground sites and research aircraft. The payloads on research aircraft consisted of several in situ instruments that differed minimally between campaigns. Ground-based trace gas observations included in situ surface and remote sensing Pandora measurements (Herman et al., 2009).

**Figure 1.**
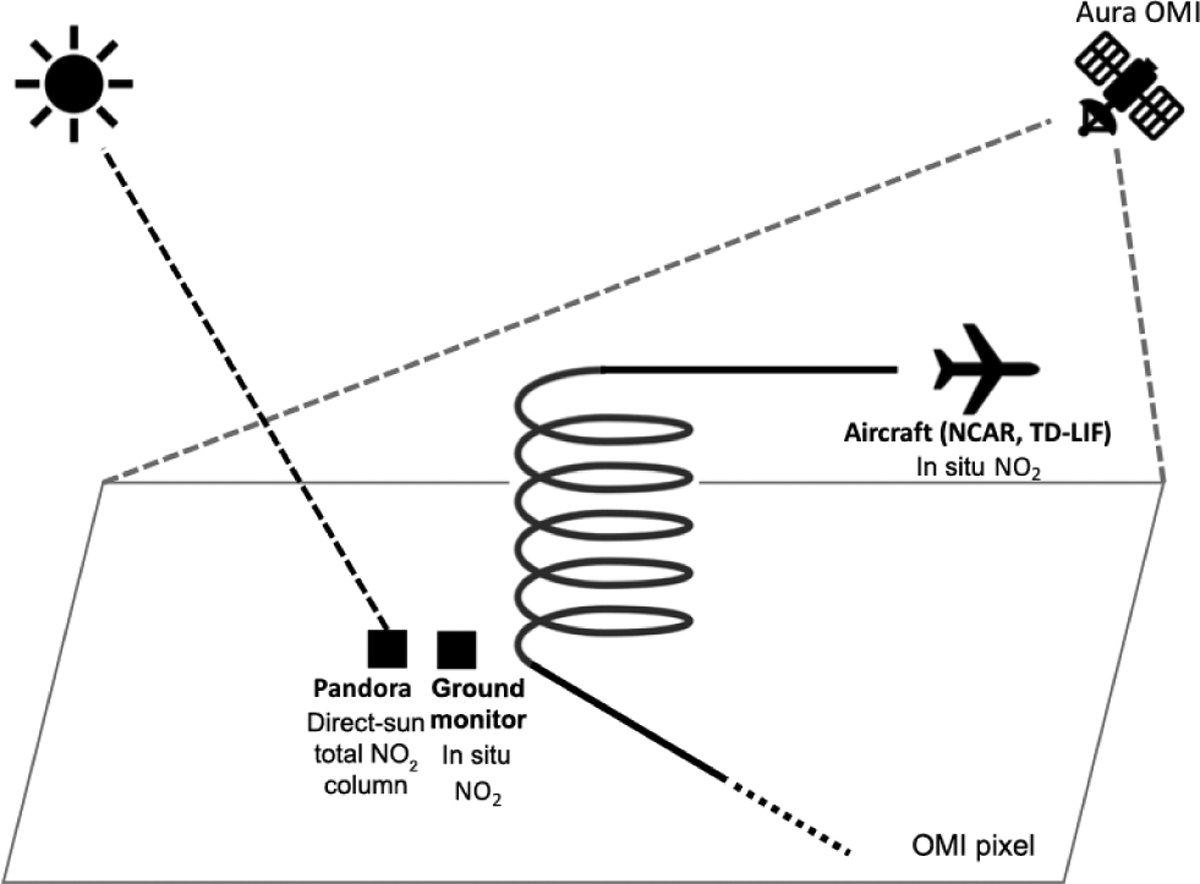
Conceptual illustration of NO_2_ observations during the DISCOVER-AQ and KORUS-AQ field campaigns. The instruments used include ground-based monitors measuring in situ NO_2_ volume mixing ratios, Pandora making direct-sun measurements to retrieve the total column NO_2_, airborne instruments measuring in situ NO_2_ profiles, and the Ozone Monitoring Instrument (OMI) aboard the Aura spacecraft reporting total column and tropospheric column NO_2_.

Figure 1 illustrates a conceptual view of the instruments and their sampling methods with their areal coverage for NO_2_ observations. While the aircraft (P-3B for DISCOVER-AQ and DC-8 for KORUS-AQ) make spirals (P-3B) or ascents and descents (DC-8) over the site, the onboard National Center for Atmospheric Research (NCAR) and thermal dissociation laser-induced florescence (TD-LIF) instruments measure in situ NO_2_ profiles. The aircraft usually visit each site two to four times a day to observe the diurnal variations of the NO_2_ profiles. The P-3B aircraft made spirals of ~ 4 km diameter, whereas the DC-8 ascents and descents covered 10–20 km. Consequently, the distance between the ground and aircraft locations was 0–5 km during the DISCOVER-AQ and 10–20 km during the KORUS-AQ campaign. Pandora and NO_2_ ground monitor instruments are typically located at ground stations close to the aircraft profiles. Throughout the day, Pandora reports the total column NO_2_ from direct-sun measurements, and the ground monitor reports the in situ surface NO_2_ mixing ratio. Finally, OMI retrievals report a tropospheric column NO_2_ once a day in the afternoon; the OMI pixel has a much larger ground footprint compared with the in situ and Pandora measurements. Table 2 lists the sites with ground-based NO_2_ monitors used in this analysis, along with the type of instrument employed at each site and the numbers of aircraft profiles and Pandora measurements available from each site near the time of OMI overpass. Detailed data descriptions follow in this section.

#### 2.1.1 Vertical distribution of NO_2_ by aircraft

In situ NO_2_ volume mixing ratios (VMRs) were measured from the NASA P-3B (DISCOVER-AQ) and DC-8 (KORUS-AQ) aircraft. The number of flights varied between campaigns, ranging from 10 for Texas to 22 for Korea. Flights took place during a range of conditions, e.g., pollution episodes, clean days, weekdays, and weekends. Measurements usually commenced in the morning and continued throughout the day with multiple sorties on a given day. During each sortie, the aircraft made vertical spirals over surface sites, sampling NO_2_ between ~ 300 m and 5 km from the Earth’s surface. In Maryland, spirals were also made over the Chesapeake Bay area, which did not have any ground monitors.

Airborne measurements were carried out using two different instruments and measurement techniques. The four-channel chemiluminescence instrument from the National Center for Atmospheric Research (NCAR) measured NO_2_ by the photolysis of NO_2_ and subsequent chemiluminescence detection of NO_2_ following the oxidation of the photolysis product NO with ozone (Ridley and Grahek, 1990). This instrument has an NO_2_ measurement uncertainty of 10 % and a 1 s, 2*σ* detection limit of 50 parts per trillion by volume (pptv). We hereafter refer to these NO_2_ measurements as “NCAR”. The thermal dissociation laser-induced florescence (TD-LIF) method used by the University of Berkeley detects NO_2_ directly and other nitrogen species (e.g., total peroxynitrates, alkyl nitrates, HNO_3_) following the thermal dissociation of all oxides of nitrogen (NO_*y*_) to NO_2_ (Thornton et al., 2000). The laser-induced fluorescence method is highly sensitive for measuring NO_2_, with a detection limit of 30 pptv. The measurement uncertainty is 5 %. This instrument has a lower NO_2_ sampling frequency than the NCAR instrument due to its alternating measurement cycle for different species. We refer to these NO_2_ measurements as TD-LIF.

**Figure 2.**
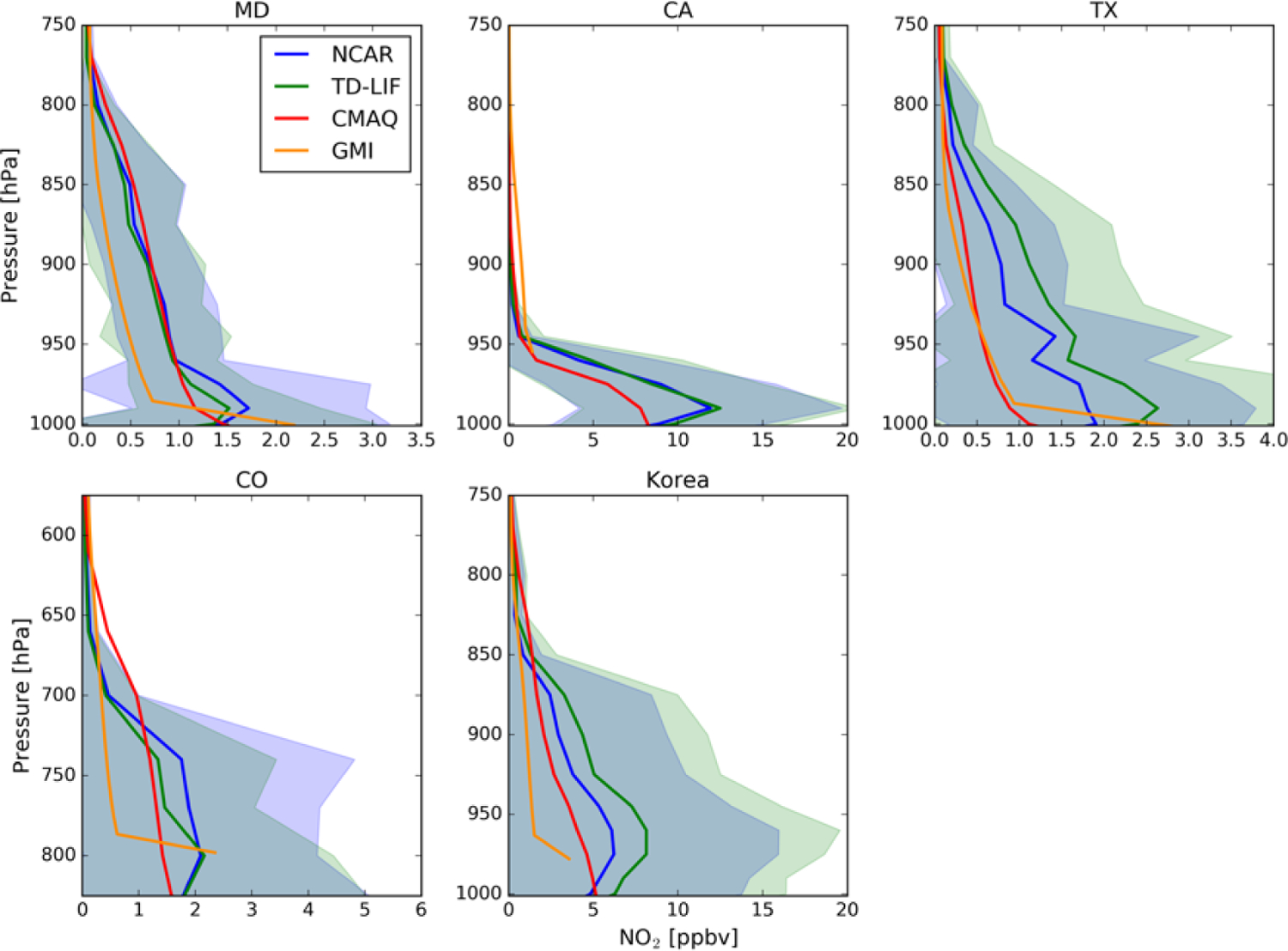
Mean early afternoon NO_2_ profiles, both observed and modeled, for the DISCOVER-AQ and KORUS-AQ campaigns. Colored lines represent the average for airborne in situ profiles from NCAR (blue) and TD-LIF (green) instruments compared with simulated profiles from the GMI global model (orange) and the CMAQ (DISCOVER-AQ) or WRF-Chem (KORUS-AQ) regional models (red). The standard deviations of airborne profiles are indicated as shaded areas for NCAR (lavender) and TD-LIF (green) instruments. The blue–gray color represents the overlap of the two.

Here we use 1 s merged data provided in the campaign data archives and focus on early afternoon measurements made within 1.5 h of the OMI overpass time (13:45 approximately). This time window of ±1.5 h is selected to maximize the number of samples while reducing effects from the diurnal variation of NO_2_. Figure 2 shows the mean NO_2_ profile for each of the DISCOVER-AQ and KORUS-AQ campaigns. Measurements show considerable spatiotemporal variation as well as some indication of a well-developed mixing layer, with the maximum mixing ratio near the ground. The mixing layer heights vary by region and season. For example, in the MD campaign conducted in summer, the mixing layer stretches up to 800 hPa (2 km). In contrast, the mean profiles from the CA campaign conducted in winter show a shallow mixing layer extending only up to 950 hPa (~ 700 m). Near-surface NO_2_ mixing ratios also vary by campaign location and possibly by season, with the highest near-surface NO_2_ in CA. In South Korea, the mean near-surface NO_2_ mixing ratio is not as high as in CA, but a very high (~ 5 ppbv) NO_2_ mixing ratio stretches up to 850 hPa, resulting in the greatest NO_2_ column. While the NCAR and TD-LIF mean profiles generally agree with each other in the MD, CA, and CO campaigns, they exhibit larger differences in TX and South Korea. Figure 2 also shows the nature of the variability in observed and simulated NO_2_ vertical profiles over the campaign domains. The observed differences between the model and observations arise primarily from a mismatch in both spatial and temporal sampling. The use of more restrictive collocation (spatial and temporal) applied for comparing different datasets in Sect. 3.1 and examining the air mass factor (AMF) effect in Sect. 2.3.2 would have resulted in different vertical distributions.

#### 2.1.2 In situ surface NO_2_ measurements

To extend the altitude range of the vertical profiles discussed in Sect. 2.1.1, we merge in situ aircraft profile measurements with coincident in situ surface NO_2_ measurements sampled over the duration of spirals (~ 20 min) by linearly interpolating the NO_2_ mixing ratios between the surface and the lowest aircraft altitudes. These new merged profiles contain a greater portion of the tropospheric NO_2_ column. During both the DISCOVER-AQ and KORUS-AQ campaigns, in situ surface NO_2_ monitors were deployed at several ground sites (Table 2). Measurements were carried out using one of four different types of NO_2_ monitors, including a chemiluminescence NO_*x*_ monitor equipped with either a molybdenum or photolytic converter, a cavity-attenuated phase shift (CAPS) spectrometer, and a cavity ring-down spectrometer (CRDS). The molybdenum converter analyzer measures NO_2_ indirectly by the thermal conversion of NO_2_ to NO using molybdenum and the detection of NO by chemiluminescence that results from the reaction of NO with ozone. Since the reduction process could convert not only NO_2_ but also other reactive nitrogen species, this instrument could overestimate NO_2_ concentrations (Dunlea et al., 2007; Steinbacher et al., 2007; Lamsal et al., 2008; Dickerson et al., 2019). The magnitude of interference depends on the relative concentrations of NO_2_, nitric acid, alkyl nitrates, and peroxy-acetyl nitrate, which vary spatially, diurnally, and seasonally and are difficult to quantify. Considering their use in the sections below (Sects. 2.3.2 and 3), we conducted a sensitivity study examining how 0 %–50 % biases in molybdenum converter measurements could impact tropospheric columns derived from merged (aircraft + surface) profiles. We found that the errors are usually rather small at < 6 % for various sites. Therefore, no attempt is made here to correct for the interference in these measurements, although we identify those sites in Table 2 and Fig. 6.

The operating principle of a photolytic converter analyzer is also gas-phase chemiluminescence, but the use of a photolytic converter to reduce NO_2_ to NO makes it more specific to NO_2_. As a result, this instrument provides nearly interference-free NO_2_ measurements, with the exception of nitrous acid (HONO; Ryerson et al., 2000). Measurement uncertainties for 1 h averages are expected to be ~ 10 % (Fehsenfeld et al., 1990).

The CAPS instrument detects NO_2_ by measuring absorption around 450 nm. Baseline measurements spanning minutes to hours with a source of NO_2_-free air are needed to determine NO_2_ amounts. In contrast to the chemiluminescence–molybdenum converter techniques, CAPS directly detects NO_2_. Its specificity for NO_2_ is affected by potential interference from species like glyoxal, water vapor, and ozone that absorb light within the band pass of the instrument. The detection limit is < 0.1 ppb for a 10 s measurement. NO_2_ measurements from CAPS and chemiluminescence NO_*x*_ monitors with a molybdenum converter are reported to agree to within 2 % (Kebabian et al., 2008).

A CRDS is a sensitive and compact detector that measures multiple nitrogen species including NO_2_. It employs a laser diode at 405 nm for the direct detection of NO_2_. Interferences arising from absorption by other trace gases, such as ozone and water vapor, are expected to be small. The measurement precision is 20 ppt at a 1 s time resolution and the accuracy is better than 5 %, which is primarily limited by the NO_2_ absorption cross section used in the data reduction process. The total reactive nitrogen (NO_*y*_) measured by the CRDS and chemiluminescence NO_*x*_ monitor with a molybdenum converter is found to agree to within 12 % (Wild et al., 2014).

#### 2.1.3 Pandora total column NO_2_

In addition to in situ measurements, each campaign hosted ground-based networks of Pandora instruments. Pandora is a small, commercially available sun-viewing spectrometer optimized for the detection of trace gases, including NO_2_. It measures direct solar spectra in the 280–525 nm spectral range with 0.6 nm resolution. A detailed description of the instrument’s design, operation, and retrieval method can be found in Herman et al. (2009, 2018). The NO_2_ retrieval algorithm includes (1) a direct-sun spectral fitting method similar to traditional differential optical absorption spectroscopy (DOAS) (Platt, 1994) using one measurement (or an average of several measurements) as a reference spectrum to derive relative NO_2_ slant column densities (SCDs), (2) the application of the Modified Langley Extrapolation (MLE) to derive total NO_2_ SCDs, and (3) the conversion of total NO_2_ SCDs to vertical column densities (VCDs) using the direct-sun air mass factor (AMF) as follows:

(1) VCD=SCD/AMF.

The spectral fitting is performed over the 400–440 nm window; it fits NO_2_ cross sections at 254.5 K (Vandaele et al., 1998), ozone (Brion et al., 1993), and a fourth-order smoothing polynomial, and it applies a wavelength shift and a constant offset. In clear-sky conditions, this instrument provides total NO_2_ VCD with a precision of 2.7×10^14^ and an absolute accuracy of 1.3×10^15^ molec cm^−2^ (Herman et al., 2018). Potential sources of error in NO_2_ retrievals include the calibration of raw data, the chosen reference spectrum, and the use of a fixed temperature for the NO_2_ cross section. Pandora NO_2_ data have been compared with data from direct-sun multifunction DOAS (MFDOAS) and Fourier transform ultraviolet spectrometry (UVFTS) (Herman et al., 2009) and have been found to agree within 12 %. These data are regularly used to validate satellite NO_2_ retrievals (e.g., Lamsal et al., 2014; Tzortziou et al., 2015, 2018; Ialongo et al., 2016).

Here, we use clear-sky quality-controlled (root mean square (rms) < 0.05 and errors < 0.05 DU) 80 s total column NO_2_ data averaged over the duration of each aircraft spiral. We infer tropospheric column NO_2_ by subtracting the OMI stratospheric column from the Pandora total column to compare with tropospheric NO_2_ from in situ and OMI observations.

### 2.2 NO_2_ simulations

#### 2.2.1 GMI simulation

The Global Modeling Initiative (GMI) three-dimensional chemical transport model (CTM) simulates the troposphere and stratosphere (Strahan et al., 2013) with a stratosphere–troposphere chemical mechanism (Duncan et al., 2007) updated with the latest chemical rate coefficients (Burkholder et al., 2015) and time-dependent natural and anthropogenic emissions (Strode et al., 2015). Aerosol fields are computed online with the Goddard Chemistry Aerosol Radiation and Transport (GOCART) model (Chin et al., 2014, and references therein). Tropospheric processes such as NO_*x*_ production by lightning, scavenging, and wet and dry deposition are also represented in the model. The GMI simulations used in this work were constrained with meteorology from the Modern-Era Retrospective Analysis for Research and Applications version 2 (MERRA-2) meteorolog ical fields (Gelaro et al., 2017) at 72 vertical levels from the surface to 0.01 hPa, with a resolution ranging from 150 m in the boundary layer to ~ 1 km in the upper troposphere and lower stratosphere, and at a horizontal spatial resolution of 1.25° longitude ×1.0° latitude.

GMI simulations have been evaluated in the troposphere and stratosphere. Strode et al. (2015) showed good agreement with tropospheric O_3_ and NO_*x*_ trends in the US in a 1990–2013 hindcast simulation. Strahan et al. (2016) demonstrated realistic seasonal and interannual variability of Arctic composition using comparisons to Aura MLS O_3_ and N_2_O. The simulation of NO_2_ in both the troposphere (Lamsal et al., 2014) and stratosphere (Spinei et al., 2014; Marchenko et al., 2015) has been shown to be in good agreement with independent measurements. We sample the model profile at the times and locations of airborne measurements. Figure 2 compares GMI NO_2_ profiles with collocated aircraft measurements during the DISCOVER-AQ and KORUS-AQ field campaigns. The GMI simulation generally captures the vertical distribution of NO_2_ in the free troposphere, is somewhat lower in the middle and upper parts of the mixing layer, and exhibits sharper gradients between the boundary layer and the surface. Due to the coarse spatial resolution of the GMI model, the surface pressure of the GMI profiles differs from the measurements, especially over complex terrain in CA, CO, and Korea.

#### 2.2.2 NO_2_ simulations using regional models

For each DISCOVER-AQ and KORUS-AQ deployment, a high-resolution model simulation was conducted. We use NO_2_ profiles from those simulations to examine their effect on retrievals in Sect. 2.3.2 and to downscale OMI NO_2_ retrievals in Sect. 2.3.3. Below we provide a brief description of each simulation. Information about model options for these simulations can be found in Table A1 in the Appendix. For most of the campaigns, the near-surface NO_2_ concentration and the model profile shapes agree in general with the NCAR and TD-LIF profiles. In TX, however, the CMAQ simulation shows lower mixing ratios than observations throughout the mixing layer (Fig. 2).

*MD*. The Weather Research and Forecasting (WRF) model was run (Loughner et al., 2014) from 24 May through 1 August 2011 at horizontal resolutions of 36, 12, 4, and 1.33 km with 45 vertical levels from the surface to 100 hPa with 16 levels within the lowest 2 km. Meteorological initial and boundary conditions were taken from the 12 km North American Mesoscale (NAM) model. Output from the 4 and 1.33 km WRF simulations were fed into the Community Multiscale Air Quality (CMAQ; Byun and Schere, 2005). Chemical initial and boundary conditions for the 4 km CMAQ run came from a 12 km CMAQ simulation covering the continental US, which was performed for the GEO-CAPE Regional Observing System Simulation Experiment (OSSE). The creation of the emissions used within the CMAQ simulation is described in Loughner et al. (2014) and Anderson et al. (2014). CMAQ was run with reduced mobile emissions by 50 % and an increase in the photolysis frequency of organic nitrate species based on Anderson et al. (2014).

*CA*. The coupled WRF–CMAQ modeling system (Wong et al., 2012) was run from 1 January through 28 February 2013 (2013 DISCOVER-AQ California campaign period) at horizontal resolutions of 4 and 2 km, with 35 vertical levels from the surface to 50 hPa and an average height of the middle of the lowest layer of 20 m. WRF version 3.8 and CMAQ version 5.2.1 were used in a coupled format, allowing for frequent communication between the meteorological and chemical transport models and indirect effects from aerosol loading on the meteorological calculations in WRF. Meteorological initial and boundary conditions were taken from the 12 km NAM reanalysis product from NOAA statistical and mathematical symbols. Observation nudging above the planetary boundary layer (PBL) using four-dimensional data assimilation (FDDA) was applied in WRF. Chemical initial and boundary conditions for the 4 km CMAQ simulation came from a 12 km CMAQ simulation covering the continental US, while initial and boundary conditions for the 2 km simulation were obtained from the 4 km WRF–CMAQ simulation. Emissions are based on the 2011 US National Emissions Inventory (NEI) with year-specific updates to point and mobile sources, while biogenic emissions were calculated inline in CMAQ using the Biogenic Emissions Inventory System (BEIS).

*TX*. To simulate the DISCOVER-AQ Texas campaign, a WRF model simulation was performed from 18 August through 1 October 2013, covering the entire field deployment in September 2013. The model was run at 36, 12, 4, and 1.33 km horizontal resolutions with 45 levels from the surface to 50 hPa. Meteorological initial and boundary conditions were taken from the 12 km North American Mesoscale (NAM) model. Output from the 4 and 1.33 km simulations were used to run the CMAQ model. Chemical and initial boundary conditions for the outer domain were taken from the Model for Ozone and Related chemical Tracers (MOZART) chemical transport model (CTM). Detailed information about these simulations and the emissions used can be found at http://aqrp.ceer.utexas.edu/projectinfoFY14_15/14-004/14-004FinalReport.pdf (last access: 5 September 2019).

*CO*. For the Colorado deployment, WRF was run from 9 July through 20 August 2014 at spatial resolutions of 12 km (covering the western US) and 4 km (covering Colorado). The model top was set at 50 hPa, with 37 levels in the vertical. Analysis fields from the European Centre for Medium-Range Weather Forecasts (ECMWF) were used for meteorological initial and boundary conditions. Chemical initial and boundary conditions for the outer domain were taken from Real Time Air Quality Monitoring System (RAQMS) model output. Further information about this simulation can be found at https://www.colorado.gov/airquality/tech_doc_repository.aspx?action=open&file=FRAPPE-NCAR_Final_Report_July2017.pdf (last access: 5 September 2019).

*Korea*. Air quality forecasts were performed using the Weather Research and Forecasting model (Skamarock et al., 2008) coupled to the Chemistry (WRF-Chem) (Grell et al., 2005) model to support KORUS-AQ flight planning and post-campaign analysis. The modeling domains consist of a regional domain of 20 km resolution covering major sources of transboundary pollutants affecting the Korean Peninsula: anthropogenic pollution from eastern China, dust from inner China and Mongolia, and wildfires from Siberia (Saide et al., 2014). A 4 km resolution domain was nested and covered the Korean Peninsula and surroundings, which encompassed the region where the DC-8 flights were planned and better resolved local sources. Anthropogenic emissions were developed by Konkuk University for KORUS-AQ forecasting and are described in Goldberg et al. (2019).

### 2.3 OMI NO_2_ observations

The Ozone Monitoring Instrument (OMI) aboard the NASA Aura satellite provides measurements of solar backscatter that are used to retrieve total, stratospheric, and tropospheric NO_2_ columns with a native ground resolution varying from 13km × 24km near nadir to 40km × 250km at swath edges (Levelt et al., 2006, 2018). The Aura satellite was launched on 15 July 2004 into a sun-synchronous polar orbit with a local Equator crossing time of 13:45 in the ascending node. OMI is one of the most stable UV–Vis satellite instruments providing a long-term high-resolution data record with low degradation (Dobber et al., 2008; DeLand and Marchenko, 2013; Schenkeveld et al., 2017). In the middle of 2007, an anomaly began to appear in OMI radiances in certain rows affecting all Level 2 products (Schenkeveld et al., 2017). This “row anomaly” can be easily identified, and the affected rows are discarded. We use OMI pixels with a cloud radiance fraction less than 50 % and quality flags indicating good data.

#### 2.3.1 Standard OMI NO_2_ Product

Here we use the Standard OMI NO_2_ Product (OMNO2) version 3.1, with updates from version 3.0 (Krotkov et al., 2017). The NO_2_ retrieval algorithm uses the differential optical absorption spectroscopy (DOAS) technique. The retrieval method includes (1) the determination of NO_2_ slant column density (SCD) using a DOAS spectral fit of the NO_2_ cross section from measured reflectance spectra over the 402–465 nm range; (2) the calculation of an air mass factor (AMF) that is required to convert SCD into vertical column density (VCD); and (3) a scheme to separate stratospheric and tropospheric VCDs. The AMF calculation is performed by combining NO_2_ measurement sensitivity (scattering weights) from the TOMS RADiative transfer model (TOMRAD; Dave, 1964) with the a priori relative vertical distribution (profile shape) of NO_2_ taken from the GMI CTM. Computation of scattering weights requires information on viewing and solar geometries, terrain and cloud reflectivities, terrain and cloud pressures, and cloud cover (radiative cloud fraction).

The version used here represents a significant advance over previous versions (Bucsela et al., 2006, 2013; Celarier et al., 2008; Lamsal et al., 2014). It includes an improved DOAS algorithm for retrieving slant column densities (SCDs) as discussed in Marchenko et al. (2015). The key features of the algorithm include more accurate wavelength registration between Earth radiance and solar irradiance spectra, iterative accounting of the rotational Raman scattering effect, and sequential SCD retrieval of NO_2_ and interfering species (water vapor and glyoxal). Solar irradiance reference spectra are monthly average data derived from OMI measurements instead of an OMI composite solar spectrum used in prior versions. Cloud pressure and cloud fraction are taken from an updated version of the OMCLDO2 cloud product that includes updated lookup tables and O_2_–O_2_ SCD retrieved with a temperature correction (Veefkind et al., 2016). A priori NO_2_ profiles are as discussed in Lamsal et al. (2015) and Krotkov et al. (2017) and use 1° latitude 1.25° longitude GMI model-based monthly a priori NO_2_ profiles with year-specific emissions. This retrieval version also uses more accurate information on terrain pressure that is calculated from high-resolution digital elevation model (DEM) data at 3 km resolution and GMI terrain pressure.

#### 2.3.2 Recalculation of OMI NO_2_ AMF using alternative NO_2_ profiles

NO_2_ vertical profiles, especially in the troposphere, vary strongly in both space and time. The simulated NO_2_ profiles from a global CTM (GMI) employed in the operational NO_2_ retrieval, while offering a good option at a global scale, may not sufficiently capture the distribution of NO_2_ at OMI’s ground resolution. Using precalculated scattering weights (Sw) made available in the OMNO2 product and alternative information on vertical NO_2_ profile shape (Xa), the OMI NO_2_ AMF can be readily recalculated (Lamsal et al., 2014):

(2) $${\text{AMF}}_{\text{trop}}=\frac{\sum_{\text{surface}}^{\text{tropopause}}\text{Sw}\cdot \text{Xa}}{\sum_{\text{surface}}^{\text{tropopause}}\text{Xa}},$$

where the integral from the surface to the tropopause yields the tropospheric AMF (AMF_trop_). Scattering weights vary with viewing and solar geometry, cloud–aerosol conditions, and surface reflectivity, but they are assumed to be independent of the vertical distribution of NO_2_. The typical vertical distribution of scattering weights is characterized by lower values in the troposphere due to reduced sensitivity owing to Rayleigh scattering and higher values (corresponding to a nearly geometric AMF) in the stratosphere. The AMF is therefore highly sensitive to NO_2_ profile shape in the lower troposphere.

**Figure 3.**
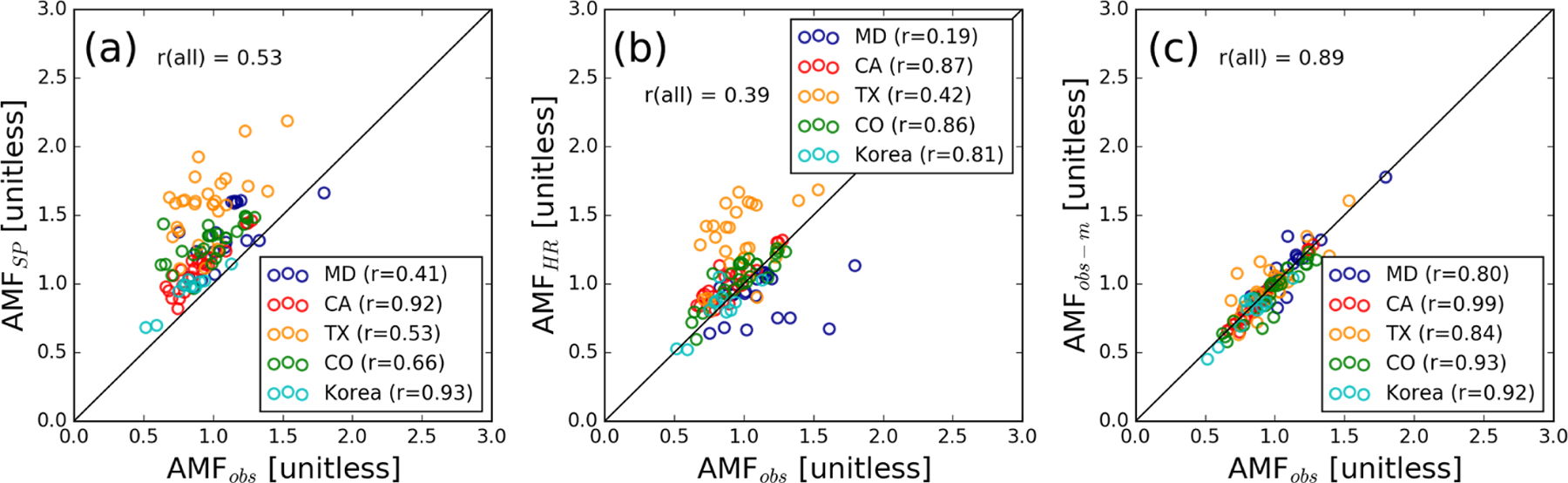
Comparison of AMFs calculated using observed NO_2_ profiles (AMF_obs_) with tropospheric AMFs in the OMI Standard Product (AMF_SP_, **a**), and those calculated using NO_2_ profiles from high-resolution model simulations (AMF_HR_, **b**). Panel (**c**) compares tropospheric AMFs using daily versus campaign-average profiles (AMF_obs-m_). The symbols are color-coded by campaign location.

Here, we investigate how a priori NO_2_ profiles affect OMI tropospheric AMF and consequently the retrieval of OMI tropospheric NO_2_ VCD. For this, we combine the measured profile (from the surface to ~ 5 km) with coincidently sampled simulated NO_2_ from GMI (5 km to the tropopause) to create a complete tropospheric NO_2_ profile. We choose the GMI simulation over the high-resolution model simulations because we found that the GMI generally better performed in the free troposphere compared to the regional models. We then interpolate the pressure-tagged NO_2_ observations (aircraft NCAR NO_2_ + surface) onto the pressure grid of the OMI NO_2_ scattering weight. The tropospheric AMFs obtained using individual measured profiles (AMF_obs_) are compared with the AMFs in the OMI Standard Product (AMF_SP_), which are calculated using the GMI yearly varying monthly climatology (Fig. 3a). AMF_SP_ is generally higher than AMF_obs_ by 34 % on average, with the largest difference (61.6 %) for TX and the smallest difference (16.6 %) for Korea; this means that the OMI SP VCDs, based on the AMF_SP_, are correspondingly smaller on average than the those based on measured profiles. The correlation ranges from fair (*r* = 0.41, *N* = 21) for MD and TX to excellent (*r* ≥ 0.92, *N* = 36) for CA and Korea, with the overall correlation coefficient of 0.53.

To explore how NO_2_ profiles from high-resolution model simulations could affect OMI NO_2_ retrievals, we calculate tropospheric AMFs using simulated monthly NO_2_ profiles (AMF_HR_). Since the OMI ground pixel size is much larger than the model grid boxes, we derive an average profile of all model grid boxes located within one OMI pixel and use it to calculate AMF_HR_. Figure 3b compares AMF_obs_ with AMF_HR_; it suggests improved agreement compared to AMF_SP_ (Fig. 3a), especially for CA, CO, and Korea, although with no significant improvement in the correlation.

We also considered how using AMFs based on monthly mean profiles, such as the OMI SP, impacts retrieved NO_2_. To assess this, we calculated AMFs using both daily (AMF_obs_) and campaign-average measured NO_2_ profiles (AMFobs-m). Figure 3c shows that AMF_obs_ and AMFobs-m agree to within 5.3 % and exhibit excellent correlation (*r* > 0.8). That is, the use of a mean profile does not make a significant difference compared to the individual daily profiles, implying that the average profile generally captures the local vertical distribution fairly well. Somewhat larger scatter in TX may be related to stronger land–sea breeze dynamics that could affect the vertical distribution of NO_2_ in both the boundary layer and free troposphere. Our results here differ from previous studies that reported improved agreement of OMI NO_2_ retrievals using simulated daily NO_2_ profiles with independent observations (Valin et al., 2013; Laughner et al., 2019), although Laughner et al. (2019) also suggested poorer performance with daily profiles in the southeast US than in other regions.

#### 2.3.3 Downscaled OMI NO_2_ data

The NO_2_ value associated with an OMI ground pixel is averaged over a large area. This spatial smoothing leads to a loss of information on sub-pixel variation, which could be considerable for NO_2_, especially over urban source regions. Therefore, it is important to recognize and address this limitation while assessing, interpreting, and using satellite NO_2_ data. Here we use high-resolution NO_2_ model simulations for sub-pixel variation.

We apply the method described by Kim et al. (2016, 2018) to downscale OMI NO_2_ retrievals, which are then compared with aircraft and Pandora data. This method applies high-resolution model-derived spatial-weighting kernels to individual OMI pixels and calculates sub-pixel variability within the pixel. The major assumption is that the model captures the spatial distribution of emission sources and NO_2_ transport patterns well. The method ensures that the quantity (total number of molecules) of the satellite data over the pixel is numerically preserved, while adding higher-resolution spatial information to the derived tropospheric NO_2_ columns.

**Figure 4.**
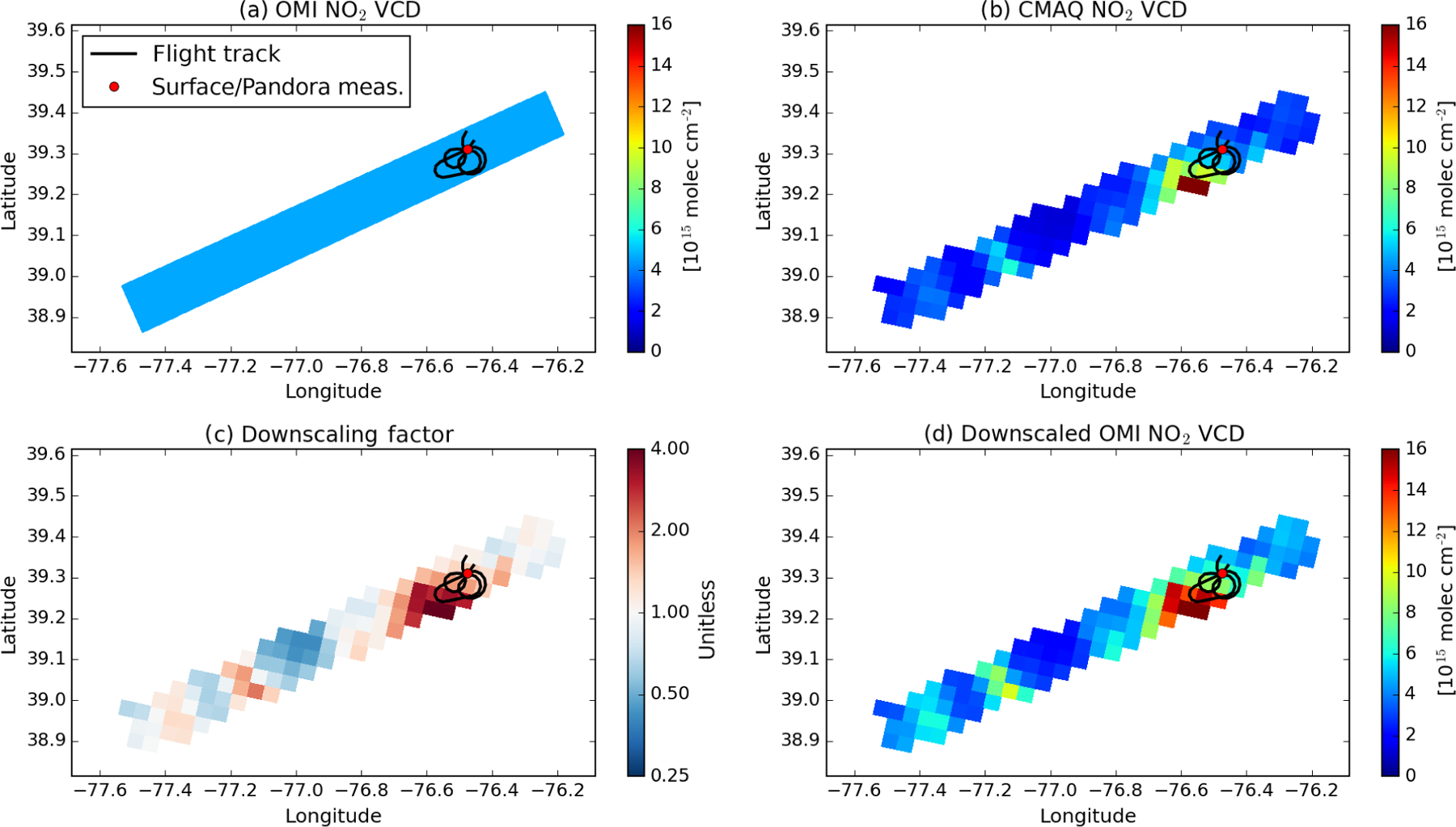
An illustration of downscaled OMI NO_2_ for an OMI pixel over Essex, MD, from orbit 37024 on 1 July 2011. Shown are the original OMI tropospheric NO_2_ VCD (**a**), coincidently sampled CMAQ NO_2_ VCD at a spatial resolution of 4×4 km2 (**b**), the spatial-weighting kernel (**c**), and downscaled OMI tropospheric NO_2_ VCD (**d**). These pixels coincide with an airborne in situ NO_2_ profile sampled during the DISCOVER-AQ Maryland campaign, and the flight route is marked with a black line. The location of the NO_2_ surface monitor and Pandora instrument is marked with a red dot.

Figure 4 illustrates the downscaling of tropospheric NO_2_ for an OMI pixel using the high-resolution CMAQ simulation over Essex, Maryland. The tropospheric NO_2_ column observed by OMI (5.9 × 10^15^ molec cm^−2^) is 25.7 % higher than the average of the CMAQ NO_2_ columns over the pixel. The spatial-weighting kernels suggest more than an order of magnitude difference in NO_2_ within this single OMI pixel. Applying the kernels to the original OMI pixel value results in a range of sub-pixel NO_2_ column values from 1.9×10^15^ over a clean background to 3.2×10^16^ molec cm^−2^ over a pol luted hot spot.

**Figure 5.**
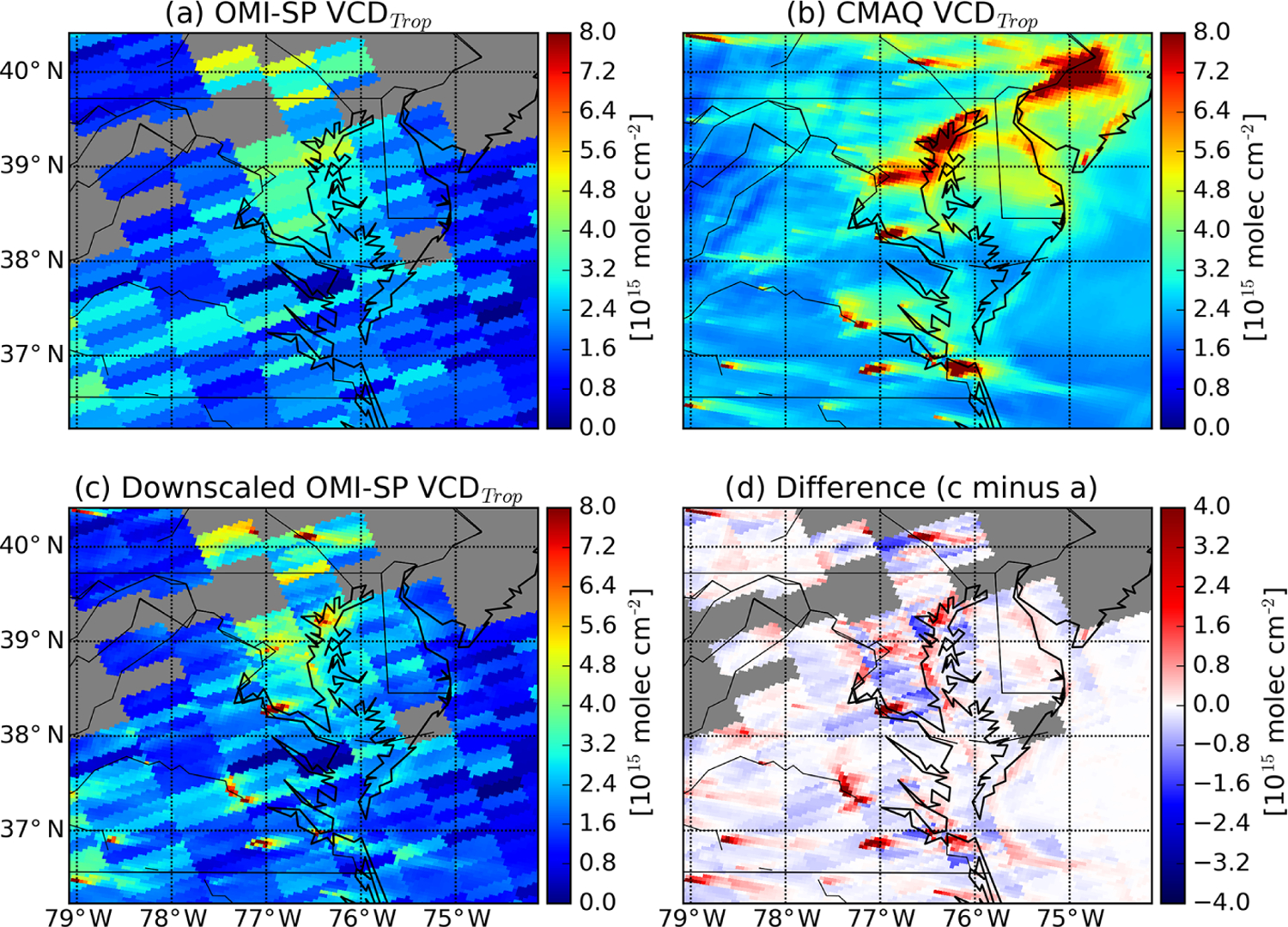
Tropospheric NO_2_ VCD maps from (**a**) OMI SP, (**b**) CMAQ, and (**c**) downscaled OMI over Maryland on 29 July 2011. Panel (**d**) shows the difference between downscaled and standard tropospheric NO_2_ VCD data (c minus a). The gray areas represent pixels with an effective cloud fraction > 0.3.

Figure 5 demonstrates how the downscaled OMI NO_2_ data using high-resolution NO_2_ output from a CMAQ simulation compare with the original OMI NO_2_ data from the standard product. Both OMI SP and CMAQ show enhanced NO_2_ columns at major urban areas, but their magnitudes differ, with OMI showing lower values. As described above, OMI’s field of view covers a large area, sampling the NO_2_ field over the entire pixel, while the actual NO_2_ distribution (better resolved by the CMAQ simulation) is defined by local source strengths, chemistry, and wind patterns that can occur at much finer spatial scales. By employing the relative ratios inside an OMI pixel rather than the overall magnitude of simulated columns, the downscaling technique yields a more detailed structure, enhancing NO_2_ over sources and dampening it elsewhere by more than a factor of 2.

## 3 Results and discussion

### 3.1 Comparison between in situ observations

**Figure 6.**
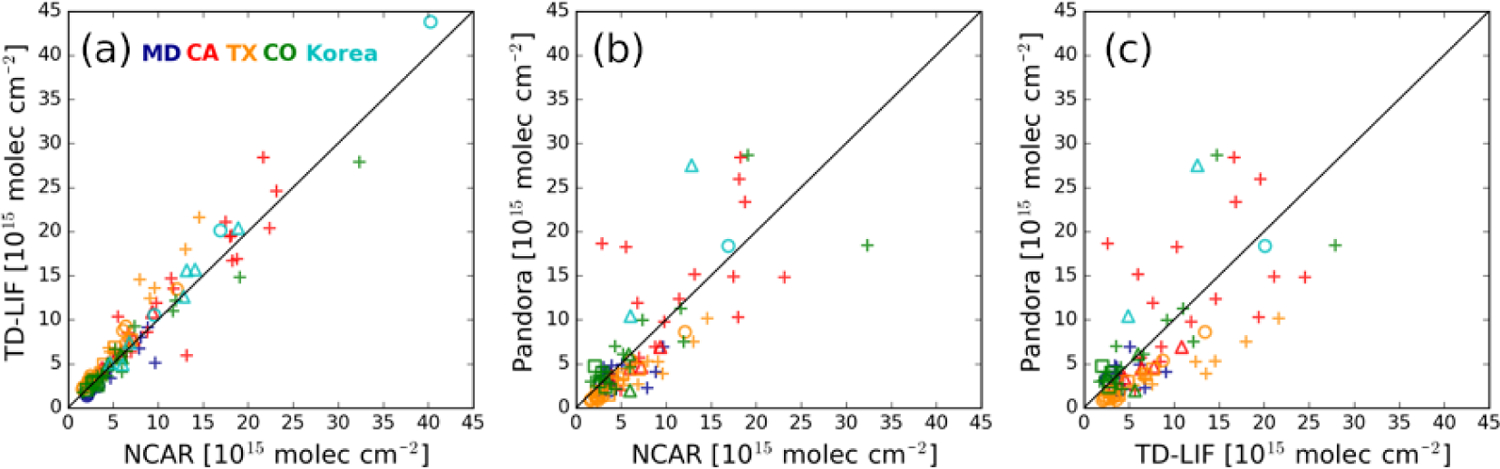
Comparison of NO_2_ tropospheric columns derived from NCAR, TD-LIF, and Pandora instruments. Different colors represent the campaign location, and the symbols represent the type of surface monitor (open circle: photolytic converter, plus: molybdenum converter, triangle: CAPS, square: CRDS).

**Figure 7.**
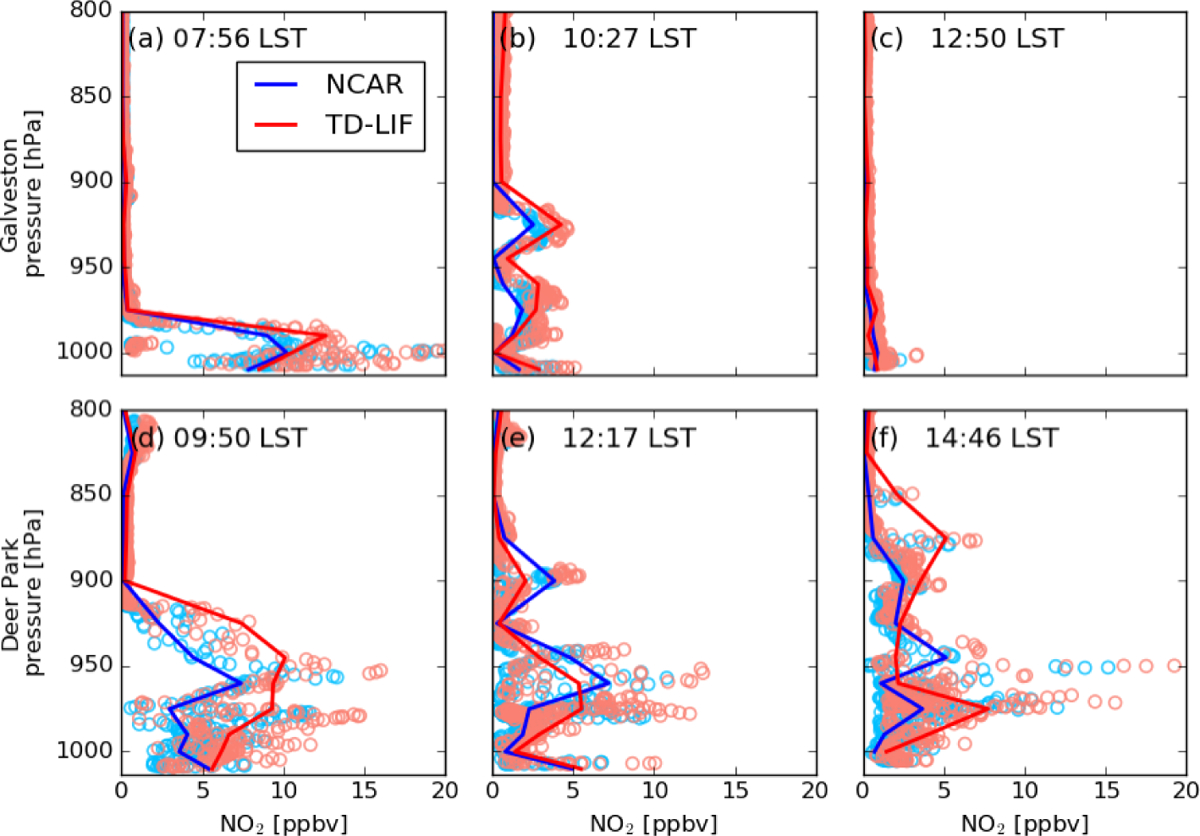
Vertical distribution of NO_2_ mixing ratios at different local solar time (LST) over Galveston (**a**, **b**, **c**) and Deer Park (**d**, **e**, **f**) in TX measured by the NCAR (light blue) and TD-LIF (orange) instruments. The circles in lighter colors represent 1 s measurements, and the solid lines show the mean values for NCAR (blue) and TD-LIF (red).

Figure 6a and Table 3 summarize how the two airborne in situ NO_2_ tropospheric column measurements compare. We derive the column amount by first extending the NCAR and TD-LIF NO_2_ profiles to the same surface NO_2_ concentration measurements and then integrating the NO_2_ profiles. The only exception is at the Chesapeake Bay during the MD campaign, the only marine site used in this study; we extend a constant NO_2_ mixing ratio measured at the lowest aircraft altitudes to the surface. To compare with OMI and Pandora retrievals, NO_2_ amounts for the missing portion from the top of the aircraft altitude to the tropopause are added from the GMI simulation. This amount varied between 4.7×10^14^ and 1.2×10^15^ molec cm^−2^ and represented an average 5 % of the tropospheric NO_2_ columns but can reach up to 50.8 % for an individual profile. Overall, the two airborne in situ columns generally agree very well and exhibit excellent correlation (*r* = 0.87–0.99). The correlation and mean difference differ among the five campaigns, with TD-LIF higher than NCAR by 31.9 % in TX and 11.6 % in Korea but lower by ~ 10% in MD and CO. The observed difference in TX is much larger than the reported uncertainty of both NCAR and TD-LIF measurements. Analysis of individual profiles suggests that the data from TD-LIF are generally higher than NCAR at all altitudes, regardless of the NO_2_ pollution level (Fig. 7). The underlying cause of this difference is not clear, but it may be associated with the applied calibration standard or an interference issue for either or both of the two measurements. The small difference elsewhere could come from the lower measurement frequency of TD-LIF compared with the NCAR instrument.

### 3.2 Comparison between Pandora and aircraft observations

Figure 6b–c and Table 3 show the comparison between Pandora and the two airborne tropospheric NO_2_ column measurements. We derive tropospheric columns from Pandora by subtracting collocated OMI stratospheric NO_2_ columns from the Pandora total column NO_2_ retrievals. The relationship between the aircraft and Pandora data is not as good as between the two aircraft measurements themselves. The use of OMI stratospheric NO_2_ columns to derive tropospheric columns from Pandora could impact the comparison between Pandora and aircraft observations; this approach is unlikely to be a significant factor over the polluted DISCOVER-AQ and KORUS-AQ campaign domains. The correlation ranges from fair (*r* = 0.42) to excellent (*r* = 0.95) for NCAR versus Pandora and poor (*r* = 0.18) to excellent (*r* = 0.94) for TD-LIF versus Pandora. The overall correlation coefficients between Pandora and the airborne NCAR and TD-LIF measurements are 0.94 and 0.91, respectively, with higher correlation in CO, TX, and Korea and lower correlation in MD and CA. Pandora data are about a factor of 2 lower than air craft measurements in TX. Elsewhere, Pandora data agree with aircraft measurements to within 20 % on average, although much larger differences are observed for individual sites. A larger discrepancy for Pandora data in TX is also reported by Nowlan et al. (2018), who used various NO_2_ measurements to evaluate GeoTASO NO_2_ retrievals. The reasons for such exceptionally large differences could include strong gradients in the NO_2_ field that are missed by aircraft spirals, errors in Pandora retrievals, or both.

### 3.3 Assessment of OMI NO_2_ retrievals

We compare OMI tropospheric NO_2_ columns with Pandora data and vertically integrated columns from aircraft spirals at 23 locations (Table 2) during the DISCOVER-AQ and KORUS-AQ field campaigns. We only analyze OMI pixels that overlap individual aircraft profiles. Spatially collocated aircraft and Pandora data are temporally matched to OMI by allowing only the measurements made within 1.5 h of the OMI overpass time. We infer tropospheric columns from Pandora by subtracting OMI-derived stratospheric NO_2_ from Pandora total columns.

**Figure 8.**
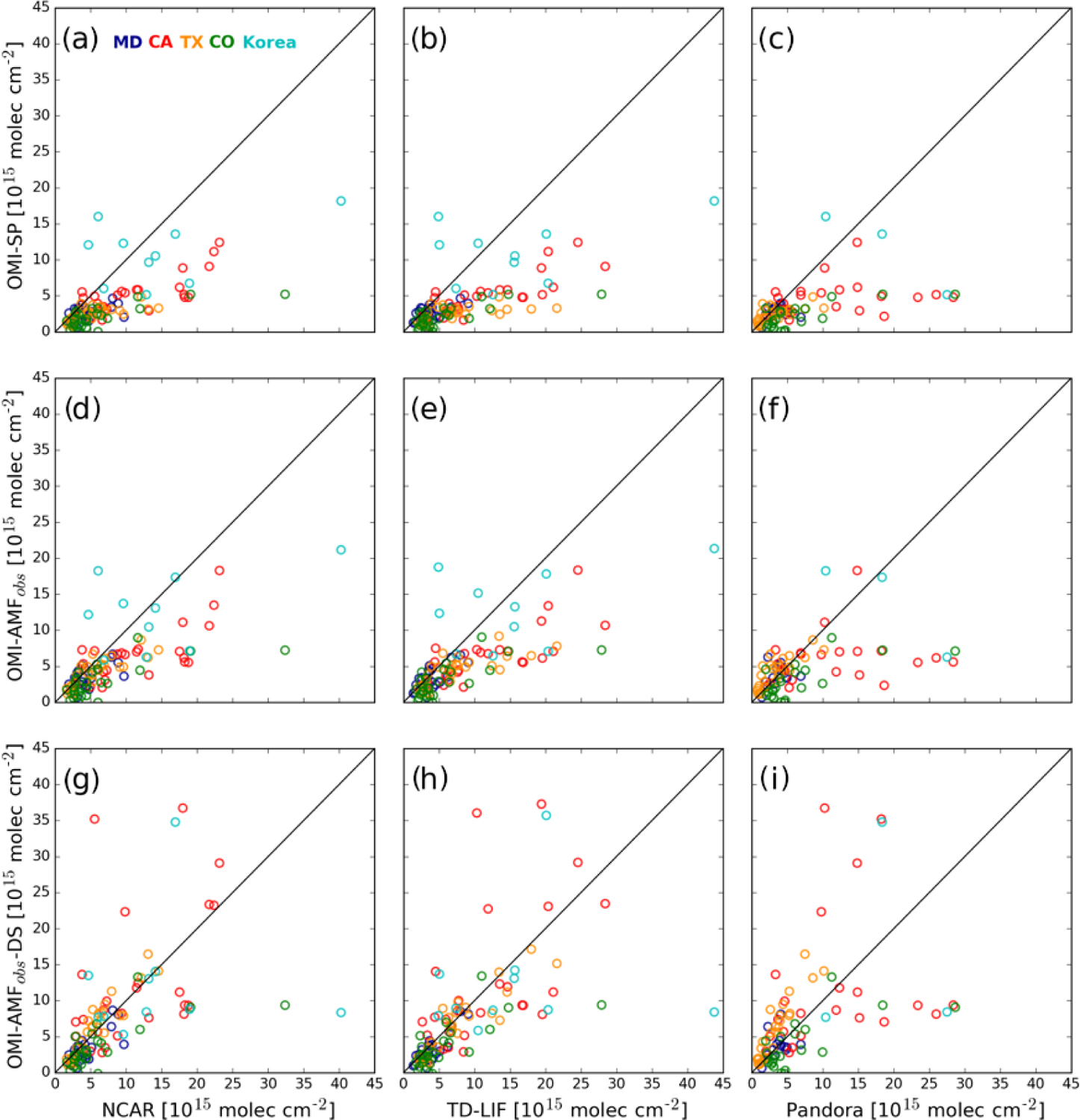
Comparison of tropospheric NO_2_ columns from OMI with the data from NCAR (**a**, **d**, **g**), TD-LIF (**b**, **e**, **h**), and Pandora (**c**, **f**, **i**) instruments. OMI retrievals are performed using the default GMI (**a**–**c**) and observed NO_2_ profiles (**d**–**i**). In addition, OMI columns in (**g**)–(**i**) are downscaled with high-resolution (CMAQ and/or WRF-Chem) model simulations. Different colors represent the campaign locations.

Figure 8a and b and Table A2 present tropospheric NO_2_ columns from the OMI Standard Product compared with integrated columns from the NCAR and TD-LIF instruments. Although the OMI and aircraft data are significantly correlated (*r* = 0.39–0.87), OMI NO_2_ retrievals are generally lower, with the largest difference in CO and the smallest difference in MD. OMI data are also lower than Pandora as shown in Fig. 8c. The magnitude of the difference and the degree of correlation with OMI vary for NCAR, TD-LIF, and Pandora measurements. This discrepancy between OMI, aircraft spiral columns, and Pandora local measurements is due to a combination of strong NO_2_ spatial variation, the size of OMI pixels, and the placement of the sites, but OMI retrieval errors arising from inaccurate information in the AMF calculation, such as a priori NO_2_ profiles, and potential errors in the validation sources themselves also contribute.

Figure 8d–f and Table A3 show the comparison after partially accounting for OMI retrieval errors arising from a priori NO_2_ profiles taken from the GMI model. Replacing the model profiles with the NCAR and TD-LIF observed NO_2_ profiles in the AMF calculations addresses the issues related to model inaccuracies, although the measured profiles may not necessarily represent the true average NO_2_ over the entire OMI pixel (e.g., Fig. 4). Nevertheless, using observed profiles reduces OMI’s mean differences with NCAR by 8 %–29.2 %, TD-LIF by 8.7 %–24.4 %, and Pandora by 6.8 %–24.2 %. Changes are largest in TX and smallest in CA and Korea. Correlations are either improved or remain similar.

Figure 8g–i and Table A4 show the comparison of OMI NO_2_ columns derived using observed profiles with NCAR, TD-LIF, and Pandora observations after accounting for spatial variation in the NO_2_ field as suggested by the CMAQ simulation. After downscaling, the agreement of OMI NO_2_ columns improves further with NCAR by 1.1 %–41.5 %, TD-LIF by 1.2 %–39.7 %, and Pandora by 1.2 %–33.2 %. The exceptions are MD for both aircraft and Pandora data and TX for Pandora data only. Changes are small in MD and Korea and large in CA and TX. The larger difference in TX is due to significant underestimation of NO_2_ by Pandora instruments. The correlation improves in MD and TX but is reduced in CA, CO, and Korea. These results suggest that downscaling helps explain some of the discrepancies between OMI, aircraft, and Pandora observations. Variations among campaign locations may also point to difficulty related to the fidelity of the CMAQ simulations.

**Figure 9.**
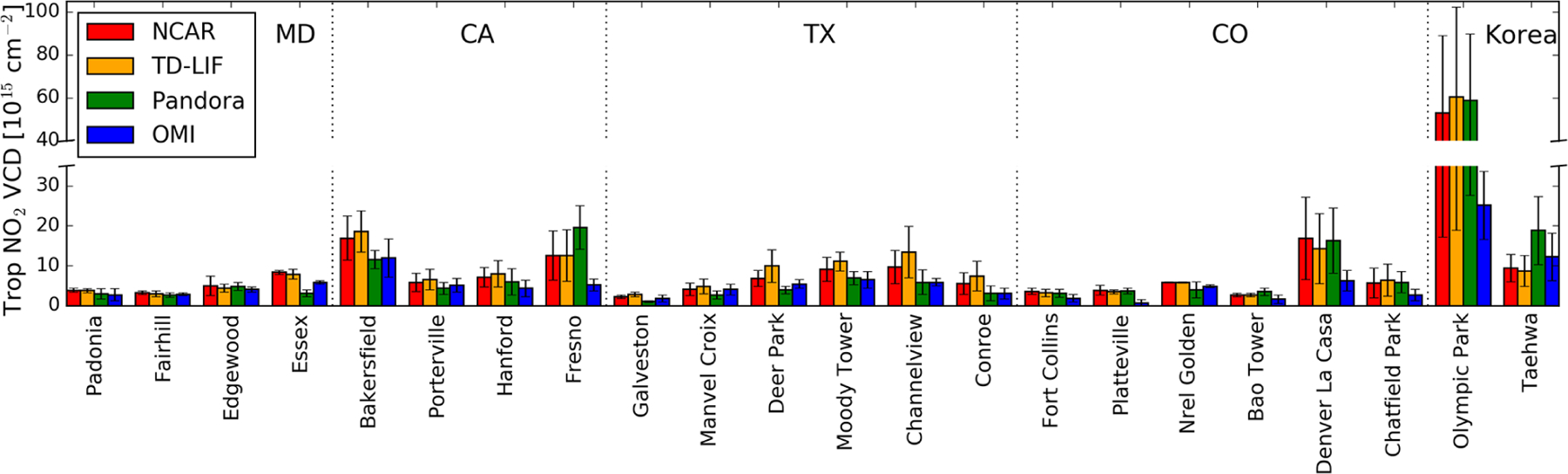
Site mean tropospheric NO_2_ VCDs calculated from NCAR (blue), TD-LIF (orange), Pandora (green), and OMI (blue). The OMI data are derived using observed NO_2_ profiles and downscaled using high-resolution model simulations. The vertical bars represent the standard deviations.

Figure 9 summarizes the comparison of OMI with aircraft and Pandora measurements. Here we present site mean columns observed from all measurements during the entire campaign periods. OMI captures the overall spatial variation in site means. In relatively cleaner places (NO_2_ VCD ≤ 5 × 10^15^ molec cm^−2^), OMI agrees well with NCAR and TD-LIF columns. OMI values are generally lower in polluted areas.

### 3.4 Implications for satellite NO_2_ validations

NO_2_ measurements from a variety of instruments and techniques taken during the DISCOVER-AQ and KORUS-AQ field deployments provided a unique opportunity to assess correlative data and realize the strengths and limitations of the various measurements. Some of the techniques are still in a state of development and evaluation, and the data have not been fully validated. Additional complications arise when comparing measurements covering different areal extents. This is particularly true for a short-lived trace gas like NO_2_ that has a large spatial gradient, especially in the boundary layer.

The NCAR and TD-LIF instruments onboard the same aircraft (P-3B during DISCOVER-AQ and DC-8 during KORUS-AQ) offer valuable insights on the vertical distribution of NO_2_, a critical piece of information needed for satellite retrievals. Despite their adjacent locations on the aircraft, they did not sample the same air mass throughout each profile due to their different NO_2_ measurement frequencies. Despite this, and even using independent measurement techniques with unique sources of uncertainties, NO_2_ measurements from the two instruments exhibit excellent correlation and very good agreement in most cases. However, varying discrepancies between the two instruments among campaigns with campaign-average differences reaching up to 31.9 % are unlikely to be related solely to the sampling issues; they are rather related to issues pertaining to measurement methods. It is crucial to reconcile these differences and improve the accuracy of these measurements for the meaningful validation and improved error characterization of satellite NO_2_ retrievals.

In situ aircraft spirals miss significant portions of the tropospheric NO_2_ column, especially from the ground to the lowest level of the aircraft altitude, typically 200–300 m above ground level. In this analysis, we account for the missing portion above the aircraft profile by using coincidently sampled simulated NO_2_ profiles. For the portion below the aircraft profile we extrapolate to surface monitor data. The latter step can be a significant error source, given that it assumes spatial homogeneity over the spiral domain. Additional errors could come from the use of different types of monitors that were deployed during the DISCOVER-AQ and KORUS-AQ campaigns (see Sect. 2.1.2). In particular, NO_2_ data from molybdenum converter analyzers are biased high by variable amounts that are difficult to quantify and correct (e.g., Lamsal et al., 2008). The use of more accurate NO_2_ monitors, such as photolytic converter analyzers, together with balloon-borne NO_2_ sondes (Sluis et al., 2010) of similar accuracy would complement in situ aircraft profiles.

While total column NO_2_ retrievals from the ground-based remote sensing Pandora instrument are useful to track temporal changes, their use for satellite validation or for comparing with aircraft spiral data can be onerous, particularly over locations with large NO_2_ spatial gradients, such as cities. Pandora’s field of view is so narrow that it serves as a point measurement. Additionally, Pandora data are subject to retrieval errors arising predominantly from the use of an incorrect reference spectrum as well as fixed temperature for the NO_2_ cross section in the spectral fitting procedure. Failure to apply a reference spectrum derived using weeks of measurements from the same site often yields systematic biases in the retrieved NO_2_ columns. Improved calibration and data processing are therefore needed to improve the Pandora data quality. Concurrent spatial NO_2_ observations from other ground-based (e.g., multi-axis differential optical absorption spectroscopy – MAX-DOAS; Vlemmix et al., 2010) or airborne (e.g., Geostationary Trace gas and Aerosol Sensor Optimization – GeoTASO; Nowlan et al., 2016; Judd et al., 2019) platforms would facilitate intercomparison among measurements of different spatial scales.

The validation of NO_2_ observations from any satellite instrument, including OMI, is complicated by a variety of factors, principally the ground area covered by the instrument’s field of view. As discussed in Sect. 3.3, disagreement between partially (spatially and temporally) matched OMI NO_2_ and validation measurements made near sources may be reasonably anticipated and ought to be expected. Therefore, it may be necessary to use a proper validation strategy, such as downscaling of satellite data using either observed or modeled NO_2_ as presented in Fig. 8g–i and Table A4. It also underscores the need for comprehensive high-quality long-term observations for validation. Enhanced agreement with OMI retrievals revised using observed NO_2_ profiles is indicative of retrieval errors from model-based a priori vertical NO_2_ profile shapes (Fig. 8d–f, Table A3) and highlights the need for approaches to address the issue. Moreover, improved accuracy in other retrieval parameters, both surface and atmospheric, helps enhance the quality of satellite NO_2_ retrievals (Laughner et al., 2019; Vasilkov et al., 2017, 2018; Lorente et al., 2018; Lin et al., 2014, 2015; Liu et al., 2019; Noguchi et al., 2014; Zhou et al., 2011)

## 4 Conclusions

We conducted a comprehensive intercomparison among various NO_2_ measurements made during the five field deployments of DISCOVER-AQ and KORUS-AQ. The field campaigns were conducted in four US states (Maryland, California, Texas, and Colorado) and South Korea. The analyzed datasets were obtained from surface monitors, the NCAR and TD-LIF airborne instruments, ground-based Pandora instruments, and the space-based OMI. We investigated the data from 23 sites among the five campaigns when measurements from all these instruments were available. We focused on an analysis of tropospheric NO_2_ column amounts. NO_2_ mixing ratio measurements from the surface monitors and airborne instruments were merged and integrated to yield tropospheric columns, while the Pandora tropospheric columns were obtained by subtracting the OMI stratospheric column from Pandora total column observations.

In order to compare OMI NO_2_ tropospheric columns with the available validation measurements, we used a combination of observed and simulated NO_2_ vertical profiles to recalculate tropospheric NO_2_ columns using the OMI Standard Product (OMNO2) version 3.1. To overcome the challenge of comparing OMI NO_2_ with its relatively large pixel size to the airborne and ground-based measurements with small spatial scales, we additionally applied a downscaling technique, whereby OMI tropospheric NO_2_ columns for each ground pixel are downscaled using high-resolution CMAQ (DISCOVER-AQ) or WRF-Chem (KORUS-AQ) model simulations. Therefore, the comparisons here include three kinds of OMI NO_2_ tropospheric columns: (1) OMI Standard Product, (2) OMI data recalculated using observed NO_2_ profiles, and (3) downscaled OMI NO_2_ data.

The tropospheric columns from the NCAR and TD-LIF airborne instruments generally show good agreement, with a mean difference of 8.4 % and correlation coefficients in the 0.87–0.99 range. The Pandora columns also agree variably with the two airborne instruments, with the campaign-average difference in the range of 3 % to 54 %, but the correlation is not as good (*r* = 0.18–0.95) as between the two airborne instruments themselves. There are differences among the campaigns. In particular, all three instruments show the largest discrepancies in the TX campaign; TD-LIF is higher than NCAR by ~ 31.9 %, and Pandora data are lower by ~ 39 % and ~ 54 % compared to NCAR and TD-LIF measurements, respectively.

All three OMI NO_2_ columns (Standard Product, based on observed NO_2_ profiles, and downscaled) exhibit good correlation with the airborne and ground-based measurements. In terms of quantitative agreement, the OMI SP column is smaller than airborne and ground-based measurements. Retrievals using observed NO_2_ profiles bring the OMI column closer to validation measurements. Applying downscaling to OMI data provides further improvement in agreement but little or insignificant change in correlation, perhaps due to the use of model simulations for downscaling.

As discussed in Sect. 3.3, disagreement between the comparatively large OMI pixel and smaller-scale ground and aircraft measurements is to be expected due to the large spatial variability of NO_2_. Techniques such as the downscaling method shown here can reduce this discrepancy. However, the robust evaluation of NO_2_ tropospheric column retrievals is further confounded by the current lack of agreement among ground-based and in-situ measurements. Future validation strategies for satellite observations of tropospheric column NO_2_ will need to address these differences.

## Figures and Tables

**Table 1.** Campaign locations and time periods.

Campaign	Location	Time period	Flight days
DISCOVER-AQ	Baltimore, Maryland	June-July 2011	14
DISCOVER-AQ	San Joaquin Valley, California	January-February 2013	11
DISCOVER-AQ	Houston, Texas	September 2013	10
DISCOVER-AQ	Denver-Ft. Collins, Colorado	July-August 2014	15
KORUS-AQ	Republic of Korea (South Korea)	May-June 2016	22

**Table 2.** Summary of ground supersites during DISCOVER-AQ and KORUS-AQ campaigns with ground-based NO_2_ measurements. The symbol *N* represents the sample size for aircraft and Pandora (in parentheses if different from that of aircraft profiles) measurements that are collocated with OMI observations. Surface NO_2_ monitors include NO_*x*_ analyzers with molybdenum converters (MCs), NO_*x*_ analyzers with photolytic converters (PCs), cavity-attenuated phase shift (CAPS) spectrometers, and cavity ring-down spectrometers (CRDSs).

Campaign	Site	Latitude, longitude	Elevation (m)	*N*	Ground monitor type
MD	Padonia	39.46° N, 76.63° W	120	6(4)	PC
	Fairhill	39.7° N, 76.86° W	109	3	PC
	Edgewood	39.4° N, 76.3° W	9	6(5)	PC
	Essex	39.31° N, 76.47° W	13	3(2)	MC
	Chesapeake^1^	39.16°N, 76.34° W^2^	-	3(0)	-
CA	Bakersfield	35.33° N, 119.0°W	117	5(3)	MC
	Porterville	36.03° N, 119.06° W	141	5	CAPS
	Hanford	36.32° N, 119.64° W	80	7(6)	MC
	Fresno	36.79° N, 119.77° W	97	8	MC
TX	Galveston	29.25° N, 94.86° W	0	7	PC
	Manvel Croix	29.52° N, 95.39° W	18	6	CRDS
	Deer Park	29.67° N, 95.13° W	6	4	MC
	Moody Tower	29.72° N, 95.34° W	64	4(2)	PC
	Channelview	29.80° N, 95.13° W	6	4	MC
	Conroe	30.35° N. 95.43° W	67	3	MC
CO	Fort Collins	40.59° N, 105.14° W	1577	3(2)	MC
	Platteville	40.18° N, 104.73° W	1522.5	5(4)	MC
	NREL-Golden	39.74° N, 105.18° W	1846	4(2)	CAPS
	Bao Tower	40.04° N, 105.01° W	1590	4	CRDS
	Denver La Casa	39.78° N, 105.01° W	1602	5(4)	MC
	Chatfield Park	39.53° N, 105.07° W	1675	5	MC
Korea	Olympic Park	37.52° N, 127.124°E	26	4(3)	PC
	Taehwa	37.31° N, 127.311°E	160	7(2)	CAPS

1 Only aircraft spirals were performed over this site.

2 The coordinate is approximate.

**Table 3.** Comparison between NCAR, TD-LIF, and Pandora NO_2_ observations.

Campaign	NCAR vs. TD-LIF		NCAR vs. Pandora		TD-LIF vs. Pandora	
No. of profs.	Mean diff. (%)		Mean diff. (%)		Mean diff. (%)	
(Pandora)	(TD-LIF - NCAR)	*r*	(Pandora - NCAR)	*r*	(Pandora - TD-LIF)	*r*
MD 21 (14)	−9.6	0.87	−24.5	0.42	−18.3	0.18
CA 25 (22)	7.2	0.93	11.1	0.65	4.8	0.58
TX 28 (26)	31.9	0.97	−39.1	0.94	−53.9	0.93
CO 26 (21)	−6.6	0.99	−2.8	0.81	4.2	0.78
Korea 11 (5)	11.6	0.99	20.3	0.95	7.5	0.94
All 111 (88)	8.3	0.99	−2.0	0.92	−9.8	0.90

## APPENDIX a

**Table A1.** Model options for each simulation. Note that all model options listed are for the domain used for the analysis.

	MD	CA	TX	CO	Korea
Dates	5/24/2011–8/1/2011	1/10/2013–2/28/2013	8/18/2013–10/1/2013	7/9/2014–08/20/2014	5/1/2016–5/31/2016
WRF model options					
Version	3.3	3.8	3.6.1	3.8.1	3.6.1
Model top	lOOhPa	50hPa	50hPa	50hPa	50hPa
Spatial resolution	4 km	4 km	4 km	4 km	4 km
Vertical levels	34	35	45	37	52
Radiation	LW: RRTM	LW: RRTMG	LW: RRTM	LW: RRTMG	LW: RRTM
	SW: Goddard	SW: RRTMG	SW: Goddard	SW: RRTMG	SW: Goddard
Land surface model	Noah Land Surface Model	Pleim-Xiu	Pleim-Xiu	Unified Noah Land	Unified Noah Land
	(Tewari et al., 2004)	(Pleim and Xiu, 2003)	(Pleim and Xiu, 2003)	Surface Model	Surface Model
Boundary layer	YSU (Hong et al., 2006)	ACM2 (Pleim, 2007)	ACM2 (Pleim, 2007)	YSU (Hong et al., 2006)	MJY scheme
Meteor, init. and	12 km NAM	12 km NAM	12 km NAM	NCAR ECMWF	0.25 degree GFS
bound, cond.					
CMAQ model options					WRF-Chem
Version	5.0	5.2	5.0.2	5.2 beta	3.6.1 (modified)
Coupled?	No	Yes	No	No	Yes
Chemical mechanism	Carbon Bond (CB05)	Carbon Bond (CB06, e51)	Carbon Bond (CB05)	Carbon Bond	Reduced hydrocarbon
	(Yarwood et al., 2005)		(Yarwood et al., 2005)	(CB06, r3)	(Pfisteretal.,2014)
Aerosol	AE5	AERO6	AE5	AERO6	MOSAIC 4 bin
Chem. init. and	12 km CMAQ v5.0	12kmCMAQv5.2	MOZART	RAQMS	24 km MACC for
bound, cond.	simulation	simulation	(outer domain)	(outer domain)	chemistry
Emissions	Described in	4 km 2013 emissions, emissions	2012 TCEQ anthropogenic	Described in report	Described in
	Loughner et al. (2014)	based on the 2011	emissions Biogenic Emission		Goldberg et al. (2019)^1^
		NEI with year-specific updates	Inventory System (BEIS)		and Saide et al. (2019)
		to EGU point sources	calculated within		
		(CEMs data),	CMAQ		
		fires and mobile			
		(MOBILE6)			

LW: longwave, SW: shortwave, RRTM: Rapid Radiative Transfer Model, RRTMG: Rapid Radiative Transfer Model for General Circulation Models, AE5: aerosols with aqueous extensions version 5, MOZART: Model for OZone and Related chemical Tracers, RAQMS: Real Time Air Quality Monitoring System, MACC: Monitoring Atmospheric Composition and Climate.

1 https://www.colorado.gov/airquality/tech_doc_repository.aspx?action=open&file=FRAPPE-NCAR_Final_Report_July2017.pdf (last access: 5 September 2019).

**Table A2.** Summary of NO_2_ comparison between the OMI Standard Product (OMISP) and NCAR, TD-LIF, and Pandora observations. The mean difference is calculated as OMI minus observations.

Campaign	NCAR vs. OMI_SP_		TD-LIF vs. OMI_S_p		Pandora vs. OMI_S_p	
No. profs (Pandora)	Mean diff. (%)	*r*	Mean diff. (%)	*r*	Mean diff. (%)	*r*
MD 21 (14)	−40.7	0.39	−34.4	0.54	−21.8	0.21
CA 25 (22)	−53.8	0.77	−56.9	0.81	−58.5	0.24
TX 28 (26)	−54.9	0.65	−65.8	0.56	−26.9	0.65
CO 26 (21)	−67.5	0.73	−65.2	0.75	−68.2	0.72
Korea 11 (5)	−41.9	0.87	−47.9	0.87	−60.1	0.8
All 111 (88)	−51.9	0.82	−55.6	0.83	−54.6	0.84

**Table A3.** Same as A2, but for OMI using AMFobs (OMIobs).

Campaign	NCAR vs. OMI_SP_		TD-LIF vs. OMI_S_p		Pandora vs. OMI_S_p	
No. profs (Pandora)	Mean diff. (%)	*r*	Mean diff. (%)	*r*	Mean diff. (%)	*r*
MD21(14)	−23.7	0.61	−17.6	0.7	2.4	0.3
CA 25 (22)	−42.4	0.73	−45.8	0.75	−47.9	0.2
TX 28 (26)	−25.5	0.82	−41.3	0.76	21.6	0.81
CO 26 (21)	−54.2	0.7	−50.5	0.71	−55.2	0.69
Korea 11 (5)	−33.9	0.87	−39.2	0.86	−53.3	0.79
All 111 (88)	−37.5	0.82	−41.5	0.82	−39.2	0.84

**Table A4.** Same as A2, but for OMIobs with downscaling (OMIDS).

Campaign	NCAR vs. OMI_SP_		TD-LIF vs. OMI_S_p		Pandora vs. OMI_S_p	
No. profs (Pandora)	Mean diff. (%)	*r*	Mean diff. (%)	*r*	Mean diff. (%)	*r*
MD21 (14)	−24.1	0.75	−18.0	0.85	0.8	0.31
CA 25 (22)	14.2	0.47	7.6	0.56	4.6	0.22
TX 28 (26)	9.5	0.94	−13.8	0.91	78.3	0.93
CO 26 (21)	−42.4	0.7	−37.7	0.71	−42.4	0.67
Korea 11 (5)	−32.8	0.73	−38.4	0.73	−52.1	0.48
All 111 (88)	−12.5	0.65	−18.0	0.68	−12.3	0.57
